# Supplementary figures and images for: Splicing accuracy varies across human introns, tissues and age
Source: bioRxiv. 2023 Mar 30:2023.03.29.534370. Preprint. [Version 1] doi: 10.1101/2023.03.29.534370 (PMC10081249; doi:10.1101/2023.03.29.534370)

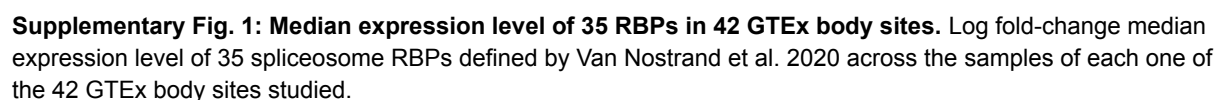

Supplement: Supplement 2 [file NIHPP2023.03.29.534370v1-supplement-2.pdf]
